# Durability of responses in patients with metastatic renal cell carcinoma treated with high dose interleukin-2 (HD IL-2)

**DOI:** 10.1186/2051-1426-3-S2-P225

**Published:** 2015-11-04

**Authors:** Joseph I Clark, Michael A Morse, Michael KK Wong, David F McDermott, Howard L Kaufman, Gregory A Daniels, Jessica C Perritt, Hong Hua, Sandra Aung

**Affiliations:** 1 Loyola University Medical Center, Division of Hematology Oncology, Maywood, IL, USA; 2 Duke University Medical Center, Durham, NC, USA; 3 University of Southern California, Los Angeles, Los Angeles, CA, USA; 4 The Cytokine Working Group; Division of Hematology/Oncology, Beth Israel Deaconess Medical Center, Boston, MA, USA; 5 Rutgers Cancer Center Institute of New Jersey, New Brunswick, NJ, USA; 6 Moores Cancer Center, University of California San Diego, La Jolla, CA, USA; 7 Prometheus Laboratories Inc., San Diego, CA, USA

## Background

HD IL-2 was FDA approved for advanced mRCC, but the data supporting its use dates to the 1990's. We designed the PROCLAIM^SM^ registry, including retrospective and prospective cohorts, to study modern outcomes and interactions with prior or subsequent targeted therapies. We now report survival analysis from the Registry and the effect of prior TT therapy.

## Methods

Inclusion criteria required patients receive at least one dose of IL-2. Survival for both cohorts (N=408) is current to March 16, 2015.

## Results

The overall response rate (ORR) and mOS are described in Table 1. In the retrospective cohort, the 1, 2, and 3 year survival rates were 89%, 69%, and 61% respectively for patients with stable disease (SD). Similarly, in the prospective cohort, 1 and 2 year survival rates for patients with SD were 95% and 76%, respectively. The mOS was not reached for patients with SD in both cohorts. There was a significant difference in mOS between SD and PD patients in the prospective cohorts, NR vs 13.9 months, p < 0.0001, there was no significant difference in mOS between SD and PD in the retrospective cohort. For patients in the retrospective and prospective cohorts who had prior targeted therapy (TT), the mOS was 15.3 and 22.1 months, respectively. Patients who did not receive TT before HD IL-2 therapy, the mOS was 48.9 months and NR, in the retrospective and prospective cohorts, respectively. There were 4 treatment-related deaths in 408 patients.

**Table 1:** 

*Updated March 16, 2015*	Retrsopective Cohort (2007-2012)N=97, 11 sites	Prospective Cohort (2011-2015)N=311, 39 sites
mOS, months	48	NR

Median follow-up, months	43.8	18.7

1,2,3 year survival rate, CP/PR	100%, 89%, 84%	100%, 85%, 79%

1,2,3 year survival rate, SD	89%, 69%, 61%	95%, 76%, n/d

ORR	20% (CR: 5%, PR: 15%)	16% (CR: 3%, PR: 13%)

CR+PR+SD	49%	55%

mOS no prioir TT/prior TT	48.9 (n=82)/15.3 (n=15)	NR (n=266)/22.1 (n=45)

## Conclusions

PROCLAIM data demonstrate that SD, previously grouped with the non-responders, has extended survival rates. TT prior to HD IL-2 therapy was associated with a lower mOS. These data support that HD IL-2 has favorable safety profile compared to data in the original package insert and remains an effective first line therapy for eligible patients with mRCC.

